# Oncogenic driver FGFR3-TACC3 is dependent on membrane trafficking and ERK signaling

**DOI:** 10.18632/oncotarget.26142

**Published:** 2018-09-28

**Authors:** Katelyn N. Nelson, April N. Meyer, Clark G. Wang, Daniel J. Donoghue

**Affiliations:** ^1^ Department of Chemistry and Biochemistry, University of California San Diego, La Jolla, California, USA; ^2^ UCSD Moores Cancer Center and University of California San Diego, La Jolla, California, USA

**Keywords:** fibroblast growth factor receptor, fusion protein, glioblastoma, FGFR3-TACC3, chromosomal translocation

## Abstract

Fusion proteins resulting from chromosomal translocations have been identified as oncogenic drivers in many cancers, allowing them to serve as potential drug targets in clinical practice. The genes encoding FGFRs, Fibroblast Growth Factor Receptors, are commonly involved in such translocations, with the FGFR3-TACC3 fusion protein frequently identified in many cancers, including glioblastoma, cervical cancer, bladder cancer, nasopharyngeal carcinoma, and lung adenocarcinoma among others. FGFR3-TACC3 retains the entire extracellular domain and most of the kinase domain of FGFR3, with its C-terminal domain fused to TACC3. We examine here the effects of targeting FGFR3-TACC3 to different subcellular localizations by appending either a nuclear localization signal (NLS) or a myristylation signal, or by deletion of the normal signal sequence. We demonstrate that the oncogenic effects of FGFR3-TACC3 require either entrance to the secretory pathway or plasma membrane localization, leading to overactivation of canonical MAPK/ERK pathways. We also examined the effects of different translocation breakpoints in FGFR3-TACC3, comparing fusion at TACC3 exon 11 with fusion at exon 8. Transformation resulting from FGFR3-TACC3 was not affected by association with the canonical TACC3-interacting proteins Aurora-A, clathrin, and ch-TOG. We have shown that kinase inhibitors for MEK (Trametinib) and FGFR (BGJ398) are effective in blocking cell transformation and MAPK pathway upregulation. The development of personalized medicines will be essential in treating patients who harbor oncogenic drivers such as FGFR3-TACC3.

## INTRODUCTION

Oncogenic driver mutations have taken a front seat in the world of cancer research. These mutations are often chromosomal rearrangements resulting in fusion proteins [1]. A recently identified fusion protein is FGFR3-TACC3, which has been discovered in glioblastoma, lung cancer, bladder cancer, oral cancer, head and neck squamous cell carcinoma, gallbladder cancer, and cervical cancer [2–4]. This fusion protein is formed by tandem duplication on chromosome 4 resulting in a fusion of the fibroblast growth factor receptor 3 (FGFR3) gene with transforming acidic coiled-coil containing protein 3 (TACC3) gene [5].

The FGFR family exhibits homologous domains of three immunoglobulin-like (Ig) domains, a transmembrane (TM) domain, and a split tyrosine kinase (TK) domain. FGFRs are activated by binding of fibroblast growth factor (FGF) ligands and heparin sulfate proteoglycans (HSPG) to the extracellular Ig-like domains [6]. This induces FGFR dimerization and activation by trans-autophosphorylation of tyrosine residues in the kinase domain activation loop. FGFR activation leads to upregulation of RAS-MAPK, PI3K-AKT, and JAK/STAT pathways. This upregulation results in cellular proliferation, migration, angiogenesis and anti-apoptosis. In cancer, oncogenic fusion proteins involving FGFRs are frequently observed, with over 40 different FGFR fusion proteins detected so far [2]. In such fusion proteins, the FGFR becomes constitutively activated by the dimerizing domain of the partner protein which brings the FGFR monomers close enough together to induce activation. In FGFR3-TACC3, the coiled-coil domain of TACC3 allows for autophosphorylation and activation of FGFR3 without the need for ligand binding [7].

TACC3 belongs to the TACC family, which provides stability of the mitotic spindle. Aurora-A phosphorylation of TACC3 results in a complex formation of TACC3, clathrin and ch-TOG. This complex localizes to the mitotic spindle microtubules and assists in their stability and cross-linking of microtubules to kinetochores. Formation of this complex is essential for mitotic spindle stability and proper cell division [8, 9]. Alteration of TACC3 expression levels has been found in many cancer types and leads to chromosomal segregation errors [10–12]. These mitotic defects can contribute to aneuploidy and cancer progression [13]. Rearrangement on chromosome 4p16, a region containing TACC3 and FGFR3, is often found in multiple myeloma. This rearrangement is often followed by increased expression of FGFR3 or TACC3 [14]. Overexpression of TACC3 is proposed as a marker for poor survival rates in multiple myeloma and breast cancer [15, 16].

It has been demonstrated that the involvement of TACC3 in the fusion protein FGFR3-TACC3 leads to an increased rate of aneuploidy and severe mitotic defects. Localization of FGFR3-TACC3 to the centrosome and mitotic spindle leads to chromosomal segregation errors and a reduction of TACC3 presence at the mitotic spindle [17, 18]. FGFR3-TACC3 has also been found to co-localize with phospho-PIN4 to induce peroxisome biogenesis and protein synthesis [19]. Although altered cellular localization and effects on mitotic defects have been well explored, it is unclear if these effects represent the initial drivers of oncogenic proliferation by FGFR3-TACC3. It has also been demonstrated that the fusion protein FGFR3-TACC3 leads to an upregulation of PI3K/AKT, STAT and MAPK pathways and pathways related to stress response and chaperone activation [2, 20, 21]. Here, we demonstrate that the oncogenic mechanism initiated by FGFR3-TACC3 is through the overactivation of canonical FGFR pathways which requires the localization of FGFR3-TACC3 to the secretory pathway and plasma membrane.

## RESULTS

### Exploring the contribution of TACC3 in FGFR3-TACC3

TACC3 has been shown to localize to spindle microtubules and centrosomes during mitosis and to the cytoplasm and nucleus during interphase [22, 23]. The presence of the C-terminal coiled-coil domain of TACC3 in the FGFR3-TACC3 fusion protein has been shown to be responsible for the localization of FGFR3-TACC3 to the nucleus and to mitotic spindle poles [7, 17]. FGFR3-TACC3 has been reported to increase the rate of aneuploidy and chromosomal separation errors, due to the presence of FGFR3-TACC3 at the mitotic spindle and the absence of TACC3 WT (wild-type) at spindle microtubules [17, 18]. These data suggest that a nuclear-localized FGFR3-TACC3 could significantly accelerate cancer progression.

**Figure 1:**
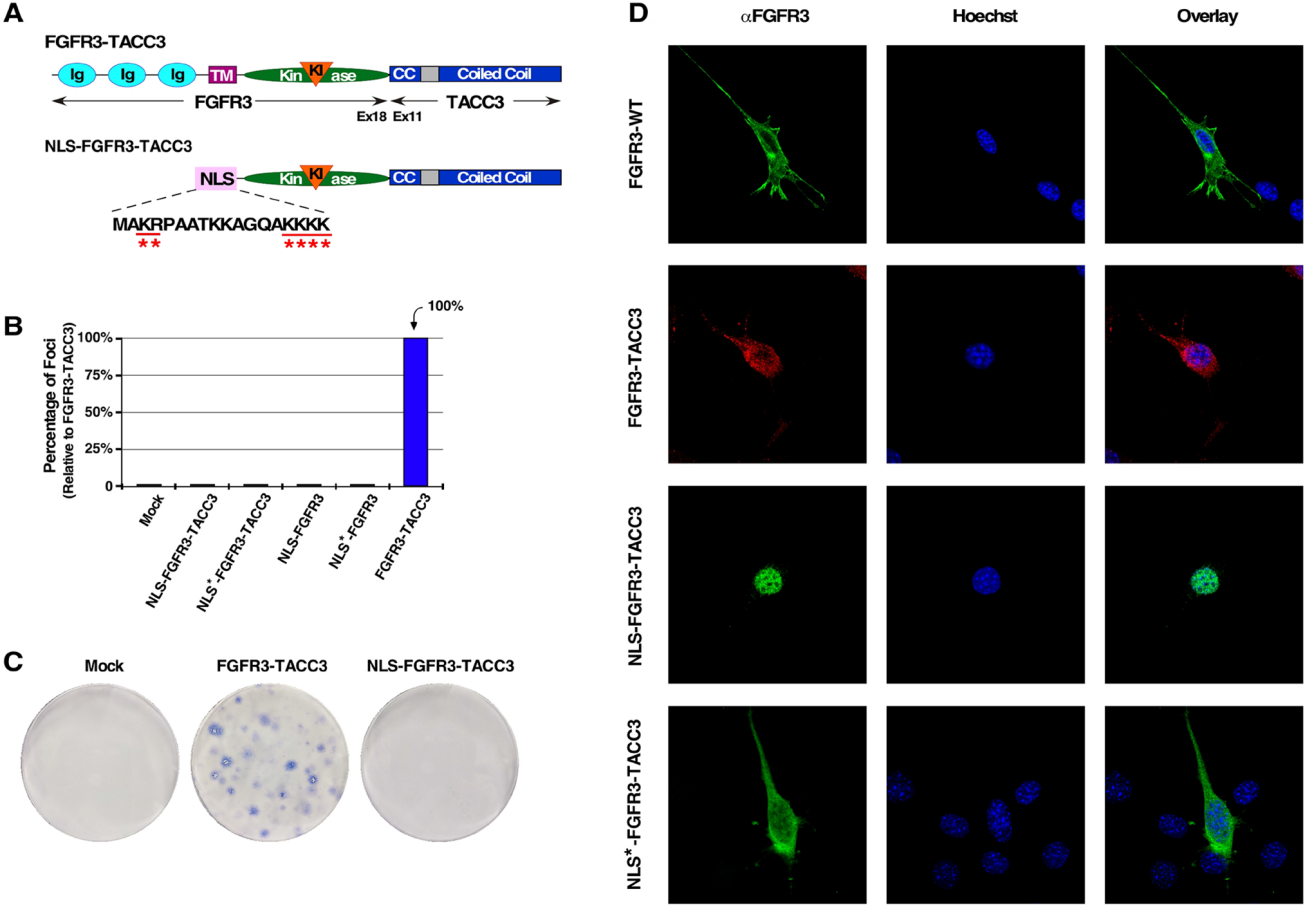
Nuclear-localized FGFR3-TACC3 does not result in cell transformation (**A**) Schematic of FGFR3-TACC3 and NLS-FGFR3-TACC3 fusion proteins. For the full length fusion, the N-terminal extracellular ligand-binding domain, transmembrane (TM), kinase, and kinase insert (KI) domains of FGFR3 are fused to the TACC3 coiled-coil (CC) domain starting at exon 11. For the nuclear-localized fusion construct, the extracellular and TM domains of FGFR3 are replaced with a bipartite Nuclear Localization Signal (NLS) derived from *Xenopus* nucleoplasmin (NLS-FGFR3-TACC3). Mutation of underlined residues to Gln (Q) results in cytoplasmic-localized FGFR3-TACC3 (NLS^*^-FGFR3-TACC3). (**B**) Transformation of NIH3T3 cells by FGFR3 and FGFR3-TACC3 derivatives. Number of foci were scored, normalized by transfection efficiency, and quantitated relative to FGFR3-TACC3 ± SEM. Assays were performed a minimum of three times per DNA construct. (**C**) Representative plates from a focus assay are shown, with transfected constructs indicated. (**D**) Representative confocal micrographs of NIH3T3 cells stably expressing the indicated constructs, using FGFR3 immunostaining directed against an intracellular kinase domain peptide of FGFR3 (P-18). Secondary antibodies were either donkey anti-goat AlexFluor488 or donkey anti-goat AlexaFluor594. Nucleus is visualized with Hoechst 33342.

To further examine the function of FGFR3-TACC3 localization, we employed a classic bipartite Nuclear Localization Signal (NLS) from *Xenopus* nucleoplasmin [24] fused in-frame with the kinase and coiled-coil domains of FGFR3-TACC3 in order to achieve nuclear localization (NLS-FGFR3-TACC3) (Figure 1A, 1D). Additionally, mutation of select positively charged residues to Gln in the NLS abrogates nuclear localization, resulting in a cytoplasmic-localized population of FGFR3-TACC3 (NLS^*^-FGFR3-TACC3) (Figure 1A, 1D). Using these populations, designed to mimic TACC3 WT behavior during interphase [23], we investigated the effects of each FGFR3-TACC3 population on oncogenicity. Surprisingly, neither the nuclear- nor cytoplasmic-targeted populations of FGFR3-TACC3 resulted in cellular transformation (data not shown), as shown by NIH3T3 focus assay (Figure 1B, 1C). This indicates that the previously identified nuclear localization of an overactivated FGFR3 receptor due to FGFR3-TACC3 fusion formation is not the driving force of NIH3T3 cell transformation. Furthering this, NIH3T3 cells transfected with both the nuclear- and cytoplasmic-targeted populations of the fusion protein (NLS-FGFR3-TACC3 and NLS^*^-FGFR3-TACC3) did not produce any cell transformation (data not shown), indicating that equal distribution between these two populations does not contribute to oncogenicity, as seen with another fusion protein NPM-ALK [25].

During interphase, the FGFR3-TACC3 fusion appears in vesicle-like structures, which is expected for a transmembrane protein and consistent with previous reports (Figure 1D) [18]. However, the addition of the TACC3 domain does alter cellular localization, as FGFR3 WT displays both cytoplasmic and plasma membrane (PM) localization (Figure 1D). While the presence of FGFR3-TACC3 may contribute to mitotic chromosomal segregation errors and aneuploidy [17, 18], this may not be the initial oncogenic driver of cellular proliferation as reflected in focus formation.

### Membrane localization is essential for FGFR3-TACC3 oncogenic activity

**Figure 2:**
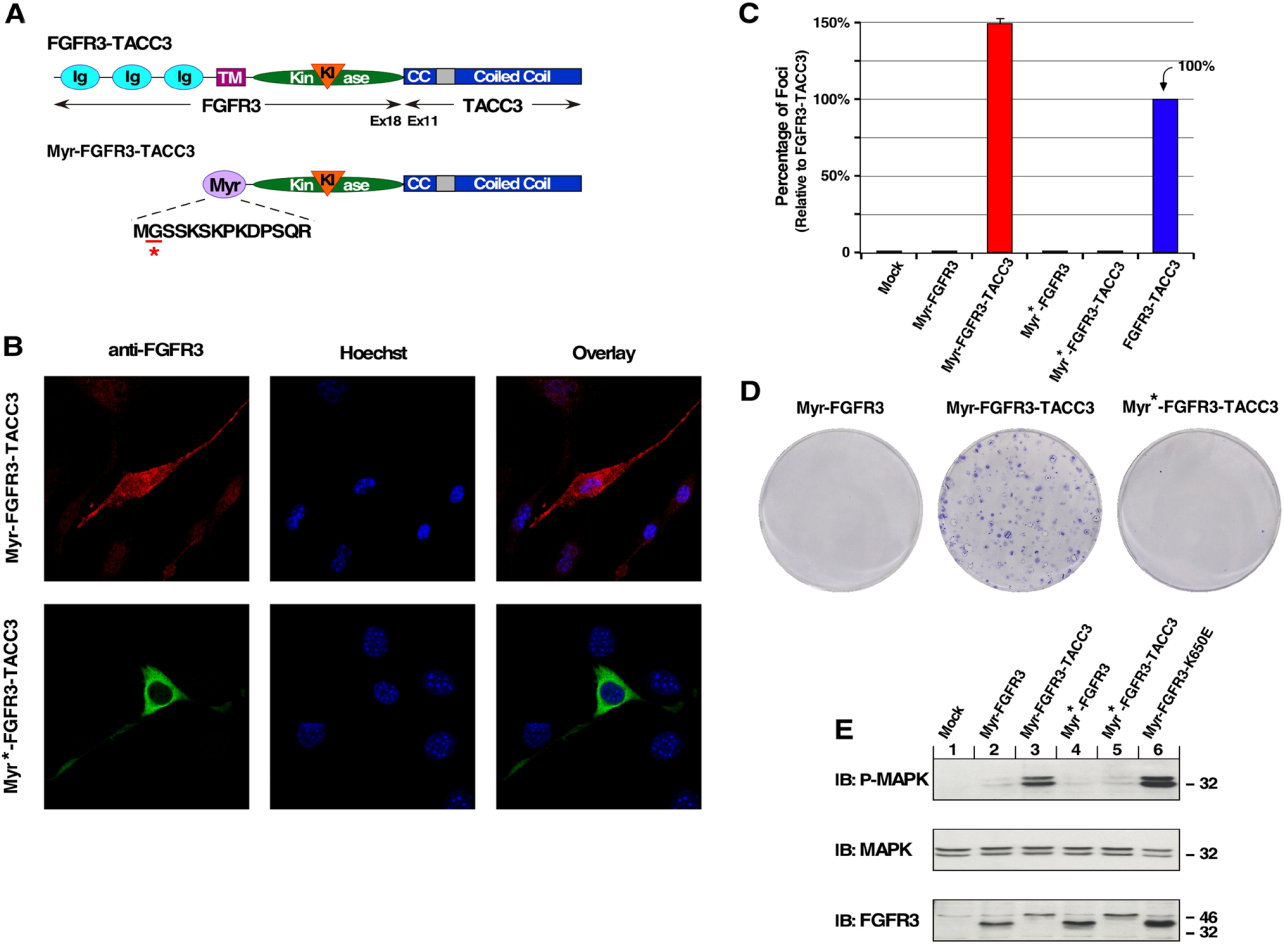
Plasma membrane-localized FGFR3-TACC3 results in cell transformation (**A**) Schematic of FGFR3-TACC3 and Myr-FGFR3-TACC3 fusion proteins. For the membrane-localized fusion construct, the extracellular and TM domains of FGFR3 are replaced with a myristylation sequence (Myr) derived from c-Src (Myr-FGFR3-TACC3). Mutation of underlined residue to Ala (A) results in cytoplasmic-localized FGFR3-TACC3 (Myr^*^-FGFR3-TACC3). (**B**) Representative confocal micrographs of NIH3T3 cells stably expressing the indicated constructs, using FGFR3 immunostaining (P-18). Secondary antibodies were either donkey anti-goat AlexFluor488 or donkey anti-goat AlexaFluor594. Nucleus is visualized with Hoechst 33342. (**C**) Transformation of NIH3T3 cells by FGFR3 and FGFR3-TACC3 derivatives. Number of foci were scored, normalized by transfection efficiency, and quantitated relative to FGFR3-TACC3 ± SEM. Assays were performed a minimum of three times per DNA construct. (**D**) Representative plates from a focus assay are shown, with transfected constructs indicated. (**E**) HEK293T cell lysates expressing FGFR3 or FGFR3-TACC3 derivatives were immunoblotted for phospho-MAPK (T202/Y204; top), MAPK (second panel), and FGFR3 (bottom).

Following our results with the NLS fusion protein, we replaced the extracellular and transmembrane domains of FGFR3 in FGFR3-TACC3 with a myristylation sequence derived from the N-terminus of c-Src (Myr-FGFR3-TACC3) [26, 27] (Figure 2A). The addition of this sequence results in N-terminal myristylation of FGFR3-TACC3; myristylation is a post-translational modification that adds myristic acid, a 14-carbon saturated fatty acid, to an N-terminal Gly residue, which directs FGFR3-TACC3 to the inner surface of the plasma membrane (Figure 2B). This membrane association represents a non-covalent type of interaction with the membrane but is distinctly different from the membrane insertion of a classic type 1 integral membrane protein such as FGFR3. FGFR3 requires an N-terminal signal sequence to direct entry into the secretory pathway, eventually reaching the cell surface after post-translational modifications such as di-sulfide bonding and glycosylation. A mutant Gly2Ala in the myristylation signal results in a non-myristylated protein that exhibits cytoplasmic localization [28] (Figure 2A, 2B). NIH3T3 cell focus assay demonstrates that only the plasma membrane-localized FGFR3-TACC3 leads to focus formation, while the cytoplasmic localized fusion protein was negative in this assay (Figure 2C, 2D).

Transfection of Myr-FGFR3-TACC3 into HEK293T cells leads to significant upregulation of the MAPK pathway, suggesting a key mechanism of cell transformation (Figure 2E). Upregulation of this pathway is consistent with previous results from our work and others which demonstrates that overactivation of MAPK but not additional downstream pathways is an important mechanism of oncogenicity and drug resistance by FGFR3-TACC3 [7, 20]. This increase in MAPK phosphorylation is comparable to Myr-FGFR3-K650E, which is a constitutively active myristylated FGFR3 produced by the mutation K650E. This mutation was originally discovered as the cause of Thanatophoric Dysplasia type II, a lethal skeletal malformation disorder [2]. Localization to the inner membrane face can produce a comparable level of cell pathway activation and transformation to FGFR3-TACC3, suggesting that the driving oncogenic force of FGFR3-TACC3 is connected to the localization of a highly active FGFR3 kinase to the membrane in order to overactivate canonical RTK pathways.

### Dual-targeted membrane associated fusion protein reinstates cell transformation

**Figure 3:**
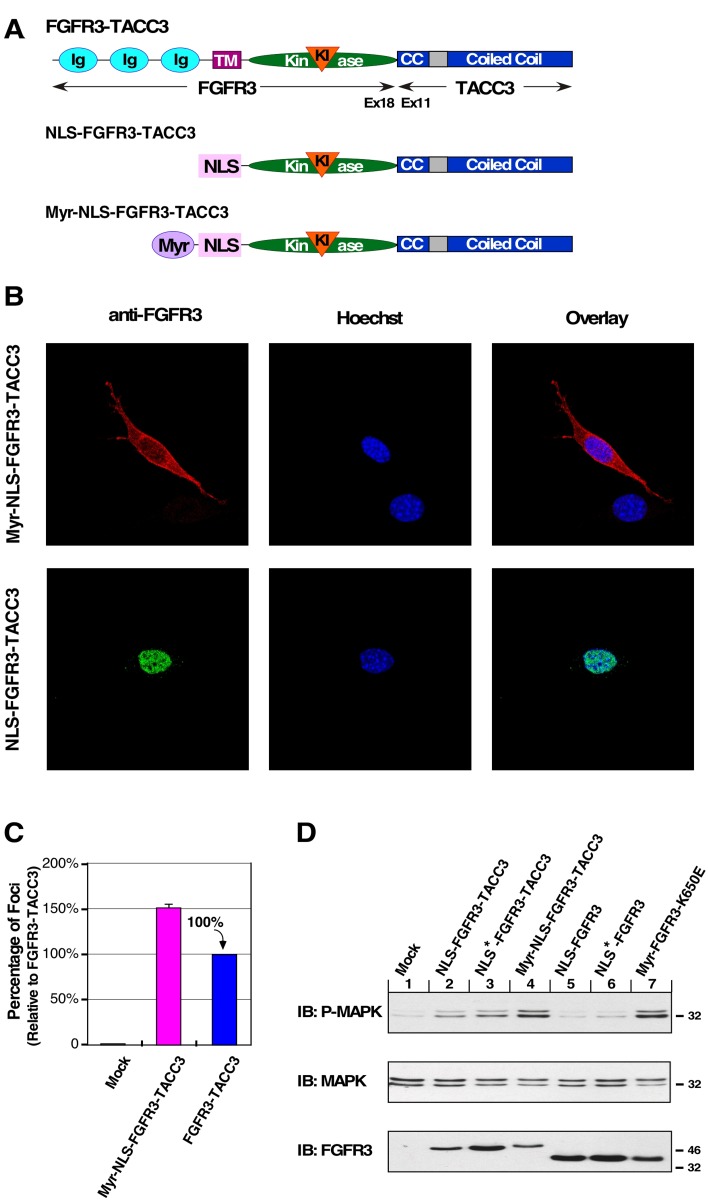
Re-localization to the plasma membrane restores oncogenic activity to NLS-FGFR3-TACC3 (**A**) Schematic of FGFR3-TACC3, NLS-FGFR3-TACC3 and Myr-NLS-FGFR3-TACC3 fusion proteins. NLS-FGFR3-TACC3 is the same fusion construct described in Fig. 1A. For the Myr-NLS derivative, the c-Src Myr sequence is fused in front of the NLS-FGFR3-TACC3 (Myr-NLS-FGFR3-TACC3). (**B**) Representative confocal micrographs of NIH3T3 cells stably expressing the indicated constructs, using FGFR3 immunostaining (P-18). Secondary antibodies were donkey anti-goat AlexFluor488 or donkey anti-goat AlexaFluor594. Nucleus is visualized with Hoechst 33342. (**C**) Transformation of NIH3T3 cells by the indicated constructs. Number of foci were scored, normalized by transfection efficiency, and quantitated relative to FGFR3-TACC3 ± SEM. Assays were performed a minimum of three times per DNA construct. (**D**) HEK293T cell lysates expressing FGFR3 or FGFR3-TACC3 derivatives were immunoblotted for phospho-MAPK (T202/Y204; top), MAPK (second panel) and FGFR3 (bottom).

To assess if oncogenic activity can be restored to the biologically inactive nuclear-localized FGFR3-TACC3, the myristylation sequence was fused in-frame 5′ of the NLS-FGFR3-TACC3 gene, creating the fusion construct Myr-NLS-FGFR3-TACC3 (Figure 3A). Our goal was to determine whether the presence of the myristylation signal would override the function of the NLS, and whether this would restore plasma membrane association and activity in cell transformation assays. Immunofluorescence analysis of this dual-targeted construct demonstrated that indeed the myristylation signal is dominant over the NLS signal, re-localizing FGFR3-TACC3 to the plasma membrane (Figure 3B). Consequently, shifting the biologically inactive NLS construct to the membrane restores biological activity and cell transformation as indicated by focus assay (Figure 3C). Also restored is the overactivation of MAPK pathway signaling by expression of Myr-NLS-FGFR3-TACC3 in HEK293T cells, furthering the connection between this pathway and cell transformation (Figure 3D). These results indicate the importance of FGFR3-TACC3 plasma membrane localization and MAPK pathway overactivation to cell transformation.

### Cell transformation is dependent on entrance to the secretory pathway

**Figure 4:**
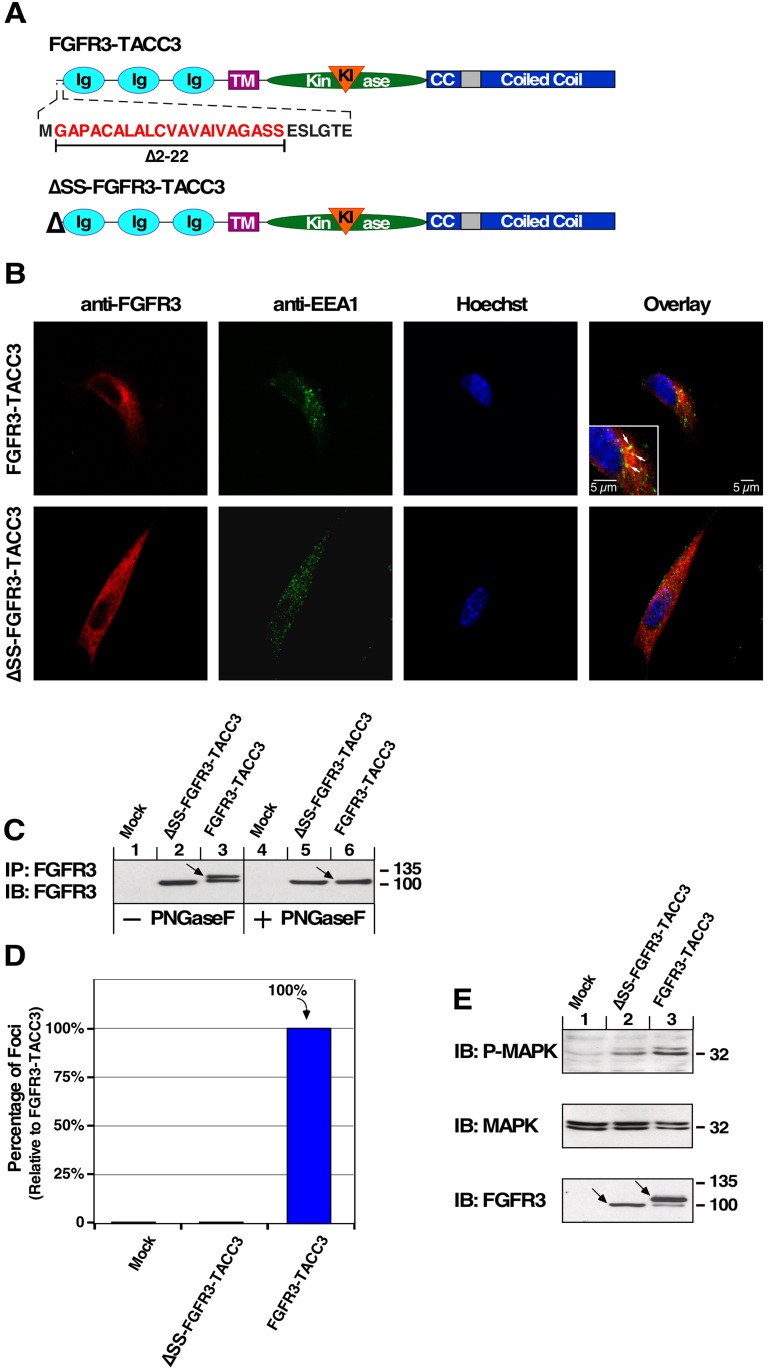
FGFR3-TACC3 depends on entrance to the secretory pathway for oncogenic effects (**A**) Schematic of FGFR3-TACC3 with detail of signal sequence. ΔSS-FGFR3-TACC3 indicates FGFR3-TACC3 with signal sequence deleted. (**B**) Confocal analysis of NIH3T3 cells stably expressing the indicated constructs reveals that FGFR3-TACC3 (red) co-localizes (yellow) with EEA1 early endosome marker (green) suggesting involvement in the secretory pathway. (**C**) HEK293T cell lysates expressing indicated constructs were immunoprecipitated with FGFR3 antibody, divided, treated with PNGase F enzyme to remove N-linked oligosaccharides, and immunoblotted with FGFR3 antibody. (**D**) Transformation of NIH3T3 cells by the indicated constructs. Number of foci were scored, normalized by transfection efficiency and quantitated relative to FGFR3-TACC3 ± SEM. Assays were performed a minimum of three times per DNA construct. (**E**) HEK293T cell lysates expressing ΔSS-FGFR3-TACC3 or FGFR3-TACC3 were immunoblotted for phospho-MAPK (T202/Y204; top), MAPK (second panel), and FGFR3 (bottom).

The appearance of FGFR3-TACC3 in vesicle-like structures (Figure 1D) could indicate its presence in secretory vesicles in transit to the membrane, consistent with type 1 integral membrane protein insertion similar to the parent protein FGFR3. To explore this, we blocked entrance of FGFR3-TACC3 to the secretory pathway by deletion of the FGFR3 signal sequence contained in the first 22 amino acids of the receptor (ΔSS-FGFR3-TACC3) [29]. The extracellular domain, transmembrane domain and kinase domain of FGFR3 and the coiled-coil domain of TACC3 remain intact (Figure 4A). The N-terminal signal peptide is homologous in the FGFR family and is responsible for targeting FGFRs for membrane insertion.

We assessed co-localization of FGFR3-TACC3 and ΔSS-FGFR3-TACC3 with a secretory pathway marker for early endosomes, EEA1, by confocal microscopy. Co-localization of FGFR3-TACC3 with this marker indicates its participation in membrane trafficking (Figure 4B, white arrows). Contrastingly, ΔSS-FGFR3-TACC3 does not co-localize with early endosomal EEA1 marker, indicating that entrance to the secretory pathway is blocked (Figure 4B). Analysis between FGFR3-TACC3 and markers for lysosomes (LAMP1), recycling endosomes (Rab11), or clathrin did not display co-localization (data not shown). As seen in Figure 4C, the multiple banding pattern of FGFR3-TACC3 (lane 3, arrow) indicates glycosylation which is not seen with ΔSS-FGFR3-TACC3 (lane 2). Treatment of immunoprecipitated FGFR3-TACC3 with PNGase F to remove N-linked oligosaccharides results in a deglycosylated form of FGFR3-TACC3 with an electrophoretic mobility pattern identical to ΔSS-FGFR3-TACC3 (Figure 4C, lanes 5 and 6). This confirms that ΔSS-FGFR3-TACC3 does not exist as a glycosylated protein and is therefore not undergoing post-translation modifications of the secretory pathway.

As determined by focus assay and immunoblot, blocking entrance to the secretory pathway also blocks focus formation, cell transformation, and MAPK pathway activation by FGFR3-TACC3. This demonstrates the need for FGFR3-TACC3 to enter the secretory pathway and undergo post-translational processing, presumably reaching the plasma membrane in order to show oncogenic effects (Figure 4D, 4E). Some detectable MAPK pathway activation by ΔSS-FGFR3-TACC3 indicates the FGFR kinase domain is able to activate this pathway in HEK293T cells, but this was not sufficient to initiate to cell transformation in NIH3T3 cells (Figure 4E). Black arrows again demonstrate FGFR3-TACC3 has a multiple banding pattern indicating different levels of post-translational processing by the secretory pathway, whereas ΔSS-FGFR3-TACC3 does not display this (Figure 4E).

### Different TACC3 breakpoints produce altered and elevated cell transformation

**Figure 5:**
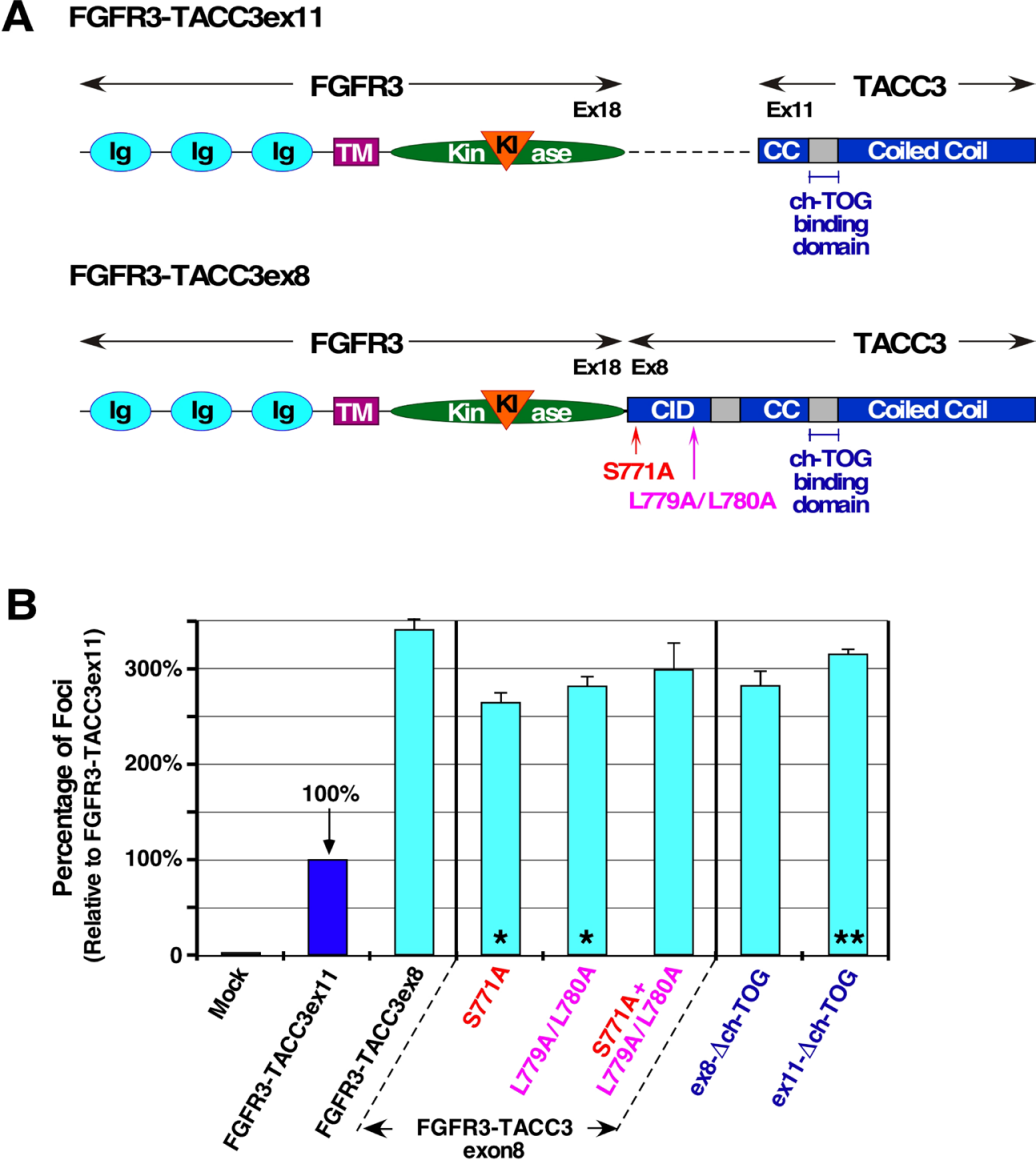
TACC3 domain mutations and their contribution to cell transformation (**A**) Schematic of FGFR3-TACC3ex11 and FGFR3-TACC3ex8 with ch-TOG binding domain indicated. Location of Aurora-A phosphorylation site (S771) and clathrin binding site (L779/L780) in FGFR3-TACC3ex8 are shown. (CID: clathrin interaction domain). (**B**) Transformation of NIH3T3 cells by the indicated constructs. Number of foci were scored, normalized by transfection efficiency, and quantitated relative to FGFR3-TACC3ex11 ± SEM. Statistical analysis by Student's *t*-test identifies significant changes in focus counts (^*^p < 0.05, ^**^p < 0.01). Assays were performed a minimum of three times per DNA construct.

FGFR3-TACC3 has been identified in human cancer with many different breakpoints between the two fused genes. Breakpoints have been found to occur within exon 16 to 19 of FGFR3 gene and within exon 4 to 11 of TACC3 [2]. The most commonly identified FGFR3-TACC3 fusion breakpoint is exon 18 of FGFR3 to exon 11 of TACC3, which this manuscript has focused on thus far. The second most common breakpoint of FGFR3-TACC3 is exon 18 of FGFR3 to exon 8 of TACC3. The introduction of the larger TACC3 gene introduces regulatory sites which are key to TACC3 WT function, including S558 Aurora-A phosphorylation site and LL566/567 clathrin binding domain, corresponding to S771 and LL779/780 in the fusion protein FGFR3-TACC3 (Figure 5A). Upon Aurora-A phosphorylation, TACC3 WT will coordinate with ch-TOG (also named CKAP5) and clathrin to form a TACC3-ch-TOG-clathrin complex to assist with mitotic spindle binding [8, 9]. Immunofluorescence shows localization of FGFR3-TACC3ex8 to be very similar to FGFR3-TACC3ex11 during interphase (data not shown). However, by focus assay, FGFR3-TACC3ex8 displays 3-fold higher cell transformation level than FGFR3-TACC3ex11 (Figure 5B). Interestingly, FGFR3-TACC3ex8 and FGFR3-TACC3ex11 display comparable levels of MAPK activation, suggesting that the increased cell transformation occurs by an additional unidentified mechanism (data not shown).

To investigate factors contributing to the difference in focus formation between the two fusion breakpoints, abrogation of Aurora-A phosphorylation site or clathrin binding site by mutation to Ala was performed (Figure 5A). While abrogation of these two sites individually did lead to a significant decrease by Student's *T* test (^*^p < 0.05), mutation of both of these sites within the same fusion protein did not lead to a significant reduction in focus formation (Figure 5B). This would indicate that association with clathrin or phosphorylation by Aurora-A via TACC3's canonical pathway does not significantly contribute to cell transformation or the oncogenic mechanism of FGFR3-TACC3.

TACC3 has been shown to interact with ch-TOG regardless of Aurora-A phosphorylation. This interaction allows the TACC3-ch-TOG complex to stabilize microtubule dynamics by binding to growing microtubule ends during interphase [30]. The binding domain of ch-TOG has been mapped to a break in the coiled-coil domain of TACC3, residues 678 to 688 in TACC3 WT. The implications of the interaction between ch-TOG and FGFR3-TACC3 have not been investigated. Previous studies have shown that deletion of the first 4 residues of this binding domain (RFEE) successfully disrupts the ch-TOG and TACC3 interaction and prevents TACC3 from localizing to growing microtubule ends [8, 30]. Deletion of these 4 residues in FGFR3-TACC3ex11 (ex11-Δch-TOG) yielded a 3-fold increase in focus formation relative to non-mutated FGFR3-TACC3ex11 (^**^p < 0.01). Contrastingly, the same deletion in FGFR3-TACC3ex8 (ex8-Δch-TOG) did not yield a significant change in the amount of foci formed (Figure 5B). This could indicate that interaction of FGFR3-TACC3ex11 and ch-TOG inhibits the ability of FGFR3-TACC3 to convey cell transformation.

### FGFR3 kinase activity and MAPK pathway upregulation are key to oncogenicity

**Figure 6:**
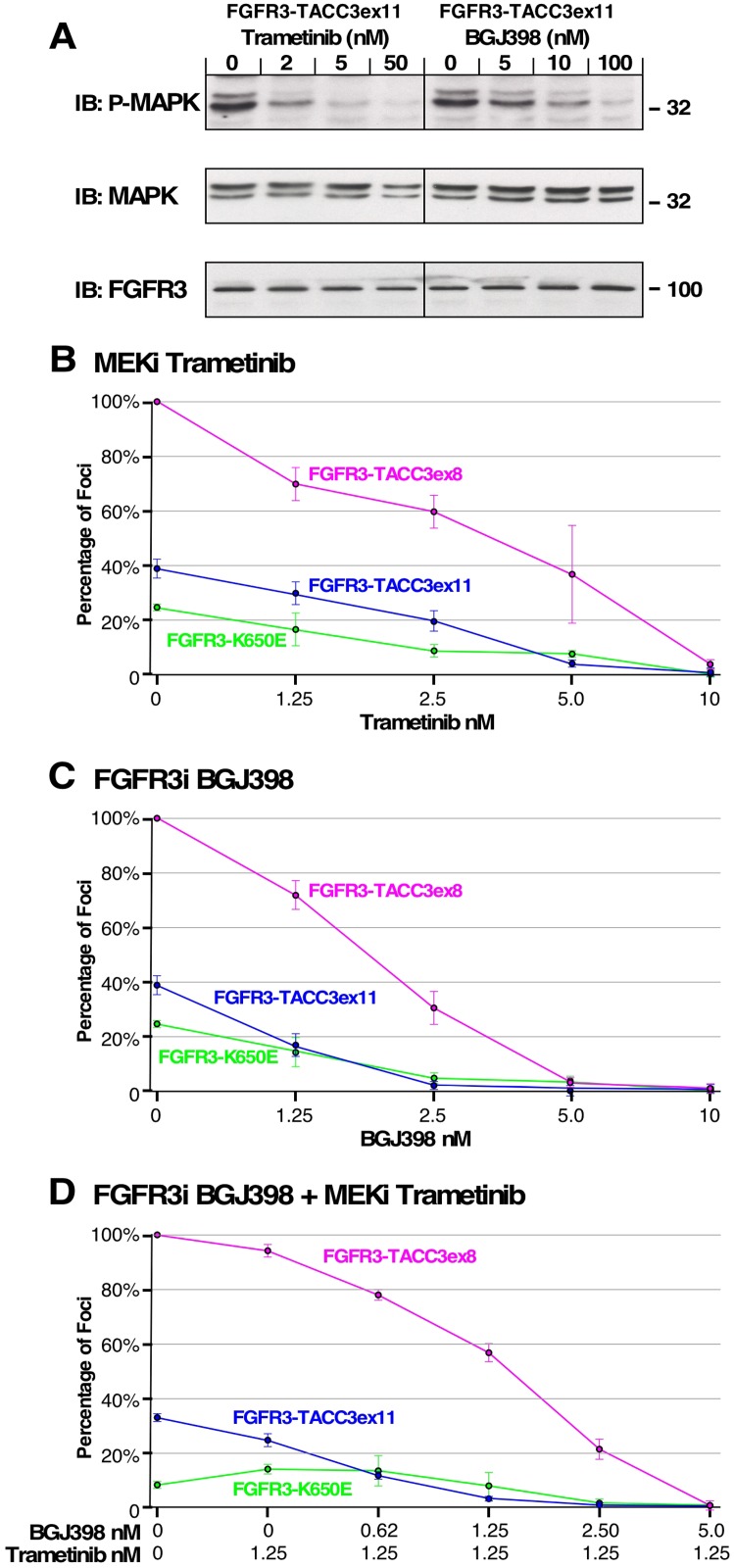
Effect of MEK and FGFR inhibitors on cell transformation and MAPK pathway signaling (**A**) HEK293T cells expressing FGFR3-TACC3ex11 were treated with Trametinib or BGJ398 at indicated concentrations and immunoblotted for phospho-MAPK (T202/Y204; top), MAPK (second panel), and FGFR3 (bottom). (**B**) Transformation of NIH3T3 cells expressing FGFR3-TACC3ex8, FGFR3-TACC3ex11 or FGFR3 K650E followed by treatment with indicated concentrations of MEK inhibitor (MEKi) Trametinib. (**C**) NIH3T3 cells expressing FGFR3-TACC3ex8, FGFR3-TACC3ex11 or FGFR3 K650E were treated with indicated concentrations of FGFR inhibitor (FGFRi) BGJ398. (**D**) NIH3T3 cells expressing indicated constructs were treated with a 1.25 nM Trametinib and varying concentrations of BGJ398. Number of foci were scored, normalized by transfection efficiency, and quantitated relative to FGFR3-TACC3ex8 ± SEM. Assays were performed three times per DNA construct.

Our data indicates that cell transformation by FGFR3-TACC3 corresponds with MAPK pathway upregulation. To examine the connection between MAPK pathway activation and focus formation, FGFR3-TACC3ex11 was transfected into HEK293T cells and treated with increasing concentrations of either FGFR kinase inhibitor BGJ398 or MEK1/2 inhibitor Trametinib (GSK1120212). Both inhibitors effectively decrease phosphorylated MAPK, as determined by immunoblot (Figure 6A).

The importance of the MAPK pathway for cell transformation is further demonstrated by NIH3T3 cell focus assay, transfected with FGFR3-TACC3ex11, FGFR3-TACC3ex8 or FGFR3 K650E activating mutation and treated with increasing concentrations of Trametinib or BGJ398 (Figure 6B, 6C). Both inhibitors individually block focus formation leading to antitumor effects, demonstrating two potential therapeutic strategies. Interestingly, differences in sensitivity to BGJ398 can be seen between the two most common breakpoints of the fusion protein, FGFR3-TACC3ex11 and FGFR3-TACC3ex8 (Figure 6C). Complete inhibition of focus formation was achieved with 2.5 nM of BGJ398 in cells transfected with FGFR3-TACC3ex11, while complete inhibition of FGFR3-TACC3ex8 required 5 nM of BGJ398 indicating that distinctive fusion breakpoints respond to the inhibitor differently. Similar effects were seen for the two fusion breakpoints treated with Trametinib (Figure 6B).

Combination of BGJ398 and Trametinib was effective in reducing cell transformation, but with less sensitivity than expected, suggesting that these inhibitors are not additive in this assay (Figure 6D). For FGFR3 K650E, both inhibitors were successful in reducing focus formation, although with less sensitivity than seen with FGFR3-TACC3ex11 and FGFR3-TACC3ex8 (Figure 6B–6D). Collectively, this data indicates a direct link between FGFR3 activation by fusion to TACC3, upregulation of the MAPK pathway, and cell transformation.

## DISCUSSION

We have determined the cellular location of FGFR3-TACC3 required to initiate cell transformation and overactivation of the canonical MAPK pathway. We have demonstrated that FGFR3-TACC3 must either enter the secretory pathway or associate with the plasma membrane to lead to oncogenic cell proliferation (Figures 1–4). Post-translational processing and plasma membrane localization is also required for the overactivation of MAPK in HEK293T cells. MAPK overactivation in HEK293T cells is only seen for FGFR3-TACC3 derivatives that induce cell transformation in NIH3T3 cells, indicating a link between this pathway and cell transformation (Figures 2, 3). The essentiality of the MAPK pathway activation to cell transformation is demonstrated by the use of Trametinib, a MEK inhibitor, which blocks cell transformation by FGFR3-TACC3 in NIH3T3 cells. FGFR inhibitor BGJ398 is also able to block focus formation indicating the FGFR kinase activity is required for cell transformation. Both inhibitors display unique levels of inhibition against different FGFR3-TACC3 fusion protein breakpoints, specifically FGFR3-TACC3ex11 and FGFR3-TACC3ex8, demonstrating the need for personalized treatment of cancers depending on the fusion breakpoint (Figure 6). Additionally, FGFR3-TACC3ex8 contains TACC3 functional sites, the Aurora-A phosphorylation site, clathrin binding site and ch-TOG binding site. However, the presence of these sites does not significantly contribute to the ability of FGFR3-TACC3ex8 to induce cell transformation (Figure 5).

The appearance of FGFR3-TACC3 in vesicle-like structures by immunofluorescence in NIH3T3 cells is an indicator of secretory vesicles in transit to the membrane and that the fusion protein is capable of being inserted in the membrane as a type 1 integral membrane protein (Figures 1, 4). Upon reaching the membrane, internalization of the fusion protein could occur quickly due to its high level of activation without the need for ligand binding. Additionally, nuclear and cytoplasmic localization of FGFR3-TACC3 does not contribute to cell transformation (Figures 1, 2). Our results indicate that in order to induce cell transformation in NIH3T3 cells, FGFR3-TACC3 must undergo post-translational processing via the secretory pathway, presumably reaching the membrane in order for cell transformation and MAPK pathway overactivation to occur. Prevention of entrance to the secretory pathway also blocks post-translational modifications, cell transformation and reduces MAPK overactivation (Figure 4). Myristylation of FGFR3-TACC3 indicates the importance of plasma membrane association for inducing cell transformation and overactivation of canonical FGFR3 pathways. Previous studies by our lab and others have identified FGFR3-TACC3-induced overactivation of MAPK and PI3K/AKT pathways, which drives cell proliferation leading to acceleration of the cell cycle and cancer progression [7, 31]. This indicates that FGFR3-TACC3 increases oncogenic proliferation by overactivation of cell signaling pathways, not by an altered localization of FGFR3-TACC3 by the TACC3 domain to the nucleus, centrosome, or mitotic spindle, as previous studies have suggested [7, 17, 18].

Our work with FGFR3-TACC3ex8 demonstrates the fusion protein's oncogenic effects are not due to mitotic involvement via TACC3's canonical pathway. Despite a significant increase in focus formation for FGFR3-TACC3ex8 compared to FGFR3-TACC3ex11, abrogation of Aurora-A phosphorylation, clathrin and ch-TOG binding sites in FGFR3-TACC3ex8 displayed no significant change in focus formation demonstrating that interaction with these proteins does not affect the biological activity of FGFR3-TACC3ex8. In FGFR3-TACC3ex11, deletion of ch-TOG binding site increases focus formation, further demonstrating that interaction with ch-TOG does not contribute to oncogenic activity (Figure 5). Interaction between FGFR3-TACC3 and ch-TOG may in fact have an inhibitory role in cell growth depending on fusion protein breakpoint. Previous studies have found that it is a removal of TACC3 from the mitotic spindle or a presence of FGFR3-TACC3 at the centrosomes that leads to chromosomal segregation errors during mitosis [17, 18]. However, incorrect cell division due to FGFR3-TACC3 does not appear to be the initial oncogenic driver in cells expressing this fusion. Consistently, studies analyzing the recruitment of other tyrosine kinases to the centrosome by fusion protein formation found centrosomal targeting to be unessential to oncogenic progression [32]. We have demonstrated that involvement with TACC3 canonical interacting proteins is not the driving force of oncogenicity.

Interestingly, we demonstrate a difference in oncogenic activity between two breakpoints of FGFR3-TACC3 (FGFR3-TACC3ex8, FGFR3-TACC3ex11) and how those breakpoints respond differently to the same inhibitor treatment. The use of kinase inhibitors stresses the importance of personalized treatment not only for knowing if a specific RTK inhibitor is useful, but also how that inhibitor affects various fusion breakpoints or cancer genotypes. We demonstrated inhibition of MEK and FGFR as two potential therapeutic strategies for cancers that harbor the FGFR3-TACC3 rearrangement. Interestingly, the two inhibitors together displayed less inhibition of cell transformation than expected. This data is also supported by inhibition of MEK and FGFR in cervical cancer cell lines to reduce cell proliferation [33]. This suggests that a different inhibitor could synergize more efficiently with an FGFR inhibitor.

Assessment of several FGFR inhibitors against FGFR3-TACC3 or other FGFR alterations in clinical trials is currently underway (https://clinicaltrials.gov/). A clinical trial enrolling patients with similar genomic alterations but various cancer types may prove useful in determining the efficacy of an inhibitor against different genomic backgrounds [33]. The fact that inhibitors display different levels of effectiveness against varied genomic backgrounds is supported not only by our work but also by those exploring inhibition of FGFR3-TACC3 in concert with PI3K inhibition [34].

This manuscript characterizes the need for FGFR3-TACC3 to be in the secretory pathway or at the cell membrane to induce cell transformation, and to activate MAPK signaling. We have shown that kinase inhibitors for MEK and FGFR are effective in blocking cell transformation and MAPK pathway upregulation. The need for precision medicine is evidenced by the different inhibitory effects these inhibitors have against various FGFR3-TACC3 breakpoints. The development of such personalized medicines will be essential in treating patients who harbor oncogenic drivers such as FGFR3-TACC3.

## MATERIALS AND METHODS

### DNA constructs

FGFR3-TACC3 gene was constructed as previously described [7]. For derivation of plasma membrane- and nuclear-localizing constructs, myristoylation signal from c-Src or nuclear localization signal from *Xenopus* nucleoplasmin was utilized as previously described [28]. Briefly, each sequence was ligated in place of the extracellular and transmembrane domains of FGFR3 resulting in fusion to residues 400 to 806 of FGFR3 or residues 400 to 953 in FGFR3-TACC3. For deletion of signal sequence of FGFR3, residues 2 to 22 were deleted by site-directed mutagenesis protocol. Deletion of ch-TOG domain followed the same protocol and deleted TACC3 residues RFEE, 792-795 in FGFR3-TACC3 or 678-681 in TACC3 [35]. Aurora-A and clathrin mutations were achieved by Quikchange site-directed mutagenesis.

### Cell culture

HEK293T cells were cultured in 10% FBS DMEM plus 1% penicillin/streptomycin in 10% CO2 at 37° C. NIH3T3 cells were maintained in 10% CS DMEM and 1% penicillin/streptomycin in 10% CO2 at 37° C.

### Antibodies and reagents

Antibodies were purchased from: FGFR3 (B-9), FGFR3 (P18) from Santa Cruz Biotechnology (Dallas, TX, USA); FGFR3 (OAAB11172) from Aviva Systems Biology (San Diego, CA, USA); phospho-p44/42 MAPK (ERK 1/2; T202/Y204; D13.14.4E), p44/42 MAPK (ERK 1/2, 9102) from Cell Signaling Technology (Danvers, MA, USA); EEA1 (610456) from BD Biosciences (San Jose, CA, USA); Alexa Fluor 488 donkey anti-goat (A11055), Alexa Fluor 594 donkey anti-goat (A11058), Alexa Fluor 488 donkey anti-mouse (A21202) from Invitrogen (Carlsbad, CA, USA); horseradish peroxidase (HRP) anti-mouse, HRP anti-rabbit, and Enhanced Chemiluminence (ECL) reagents were from GE Healthcare (Little Chalfont, UK). Geneticin (G418) was from Gibco (Waltham, MA, USA), and Lipofectamine 2000 was from Invitrogen (Carlsbad, CA, USA). PNGase F was purchased from NEB (P0704S) and Pierce Protein A/G Magnetic Beads (88802) were purchased from Thermo Fisher (Waltham, MA, USA). Inhibitors BGJ398 (S2183) and Trametinib (S2673) were purchased from Selleckchem (Houston, TX, USA).

### Immunoprecipitation and immunoblot analysis

24 h before transfection, HEK293T cells were plated at 1 × 10^6^ cells/100-mm plate. Calcium phosphate method was used to transfect 3 μg plasmid DNA in 3% CO_2_ as described previously [7]. For immunoblot analysis, after cell starvation and collection, cells were lysed in RIPA buffer [50 mmol/l Tris-HCl (pH 8.0), 150 mmol/l NaCl, 1% TritionX-100, 0.5% sodium deoxycholate, 0.1% SDS, 50 mmol/l NaF, 1 mmol/l sodium orthovanadate, 1 mmol/l PMSF, and 10 mg/ml aprotinin]. Total protein concentration was measured using Lowry assay. For immunoprecipitation, cells were lysed in 1% NP40 Lysis Buffer [20 mmol/l Tris-HCl (pH 7.5), 137 mmol/l NaCl, 1% Nonidet P-40, 5 mmol/l EDTA, 50 mmol/l NaF, 1 mmol/l sodium orthovanadate, 1 mmol/l phenylmethylsulfonylfluoride (PMSF), and 10 mg/ml aprotinin]. Protein concentration was measured by Bradford assay. Lysates were incubated overnight with antibodies at 4° C with rocking. Complexes were collected with Pierce Protein A/G Magnetic Beads (88802) according to manufacturers protocol. For PNGase digest, PNGase F Protocol from manufacturer NEB was followed.

10% or 12.5% SDS-PAGE separated samples before transfer to Immobilon-P PVDF membranes (Millipore, Burlington, MA, USA). Membranes were blocked in 3% bovine serum albumin (BSA)/TBS/0.05% Tween 20 or 3% milk/TBS/0.05% Tween 20. Immunoblotting was completed as previously described [36].

### Immunofluorescence

Stable cell lines were created by transfecting NIH3T3 cells with Lipofectamine 2000 with FGFR3-TACC3 derivatives in pLXSN vector with Geneticin as the selectable marker. Cells were grown in 500 μg/ml G418 supplemented media for 14 days. Cell lines were created for all constructs except FGFR3 WT and ΔSS-FGFR3-TACC3. Stable cell lines were plated on 60mm plates with 6 coverslips at 1 × 10^5^ cells per plate. Coverslips were PLL coated (GG-12-1.5-PLL, Neuvitro, Vancouver, WA, USA). 24 h after plating, cells were starved with 0% serum DMEM for additional 24 h. Coverslips were fixed with 4% paraformaldehyde/PBS for 10 min.

For FGFR3 WT and ΔSS-FGFR3-TACC3, NIH3T3 cells were plated at 2 × 10^5^. 24 h after plating, cells were transfected with Lipofectamine 2000. 18–20 h after transfection, cells were refed with 10% CS DMEM for 6 h until media was changed to 0% serum DMEM for 24 h. Cells were fixed with 4% paraformaldehyde/PBS for 10 min.

For immunofluorescence staining, cells were permeabilized with 0.1% Triton X-100/PBS for 20 min, blocked with 5% BSA/PBS before incubation with primary antibodies, goat anti-FGFR3 (1:500 or 1:1500) or EEA1 (1:25). After washes, cells were treated with secondary antibodies, donkey anti-goat Alexafluor488 (1:2000), donkey anti-goat Alexafluor594 (1:1500), or donkey anti-mouse Alexafluor 488 (1:250). Nucleus is visualized with Hoechst 33342 (1 μg/ml, 15 min). Cells were examined on Leica SP5 Confocal/MultiPhoton microscope (UC San Diego Neuroscience Core Facility). Images were processed with Leica LAS Lite and FIJI software.

### Focus assay

NIH3T3 cells were plated at a density of 4 × 10^5^ cells/60-mm plates in 10% CS DMEM 24 h before transfection. Lipofectamine 2000 Reagent was used to transfect cells with 10 μg plasmid DNA. Cells were re-fed with DMEM 10% CS 22–24 h after transfection. Cells were split 1:12 onto duplicate 100-mm plates 24 h later with 2.5% CS DMEM. Cells were refed every 3–4 days. After 14 days, foci were scored, fixed with methanol, and Geimsa stained. Transfection efficiency was determined by Geneticin (G418, 0.5 mg/ml)-resistant colonies plated at 1:240 dilution. Number of foci were scored, normalized by transfection efficiency, and quantitated relative to FGFR3-TACC3 ± SEM. Assays were performed a minimum of three times per DNA construct. Statistical analysis by Student's *t*-test identifies significant changes in focus counts and a two-tailed *P*-value of 0.05 was considered significant.

For inhibitor treatment, 24 h after splitting cells 1:12 onto 100-mm plates, cells were refed with 2.5% CS DMEM containing indicated concentrations of BGJ398 or Trametinib. Cells were refed with 2.5% CS DMEM with the same inhibitor concentrations every 3–4 days. After 14 days, foci were scored, fixed with methanol, and Geimsa stained. Transfection efficiency was determined by Geneticin (G418, 0.5 mg/ml)-resistant colonies plated at 1:240 dilution.

## Abbreviations

FGFR3: Fibroblast Growth Factor Receptor 3
TACC3: Transforming Acidic Coiled-Coil Containing Protein 3
